# Genome-wide transcriptional profiling identifies molecular markers associated with early carcinogenesis in high-grade bladder cancer

**DOI:** 10.1038/s41598-026-36530-1

**Published:** 2026-03-26

**Authors:** Rifat Burak Ergul, Canan Kucukgergin, Zeynep Birsu Cincin, Bedia Cakmakoglu, Selcuk Erdem, Tzevat Tefik, Mehmet Oner Sanli, Ismet Nane, Faruk Ozcan, Sule Seckin

**Affiliations:** 1 Department of Urology, Istanbul Faculty of Medicine, Istanbul University, Istanbul, Turkey; 2 Department of Biochemistry, Istanbul Faculty of Medicine, Istanbul University, Çapa, Fatih, 34093 Istanbul, Turkey; 3 Department of Medical Biochemistry, Faculty of Medicine, Istanbul Nisantasi University, Istanbul, Turkey; 4 Department of Molecular Medicine, Aziz Sancar Experimental Medicine Research Institute, Istanbul University, Istanbul, Turkey; 5 Division of Urologic Oncology, Department of Urology, Istanbul Faculty of Medicine, Istanbul University, Istanbul, Turkey; 6 Mus State Hospital, Mus, Turkey

**Keywords:** Carcinogenesis marker, Genetic, Bladder cancer, Microarray, Molecular pathways, RNA expression, Biomarkers, Cancer, Computational biology and bioinformatics, Genetics, Molecular biology, Oncology

## Abstract

To determine genome-wide transcriptional coverage of well-characterized genes, gene candidates, and splice variants associated with invasive process on bladder carcinogenesis (BC), we investigated the whole-genome gene expression profile of high-grade Turkish BC patients. We collected high-grade bladder tumours (*n* = 12) and paired standard tissue samples (*n* = 12). To find differentially expressed genes related to bladder cancer’s metastatic pattern, we performed the human whole-genome expression profiling through the Illumina Human HT-12 Expression Beadchip system. Ingenuity Pathway Systems (IPA), i-Pathway Guide and Cytoscape software were used to determine statistically significant genes, networks, biological pathways between tumour and control groups. Cyclin A2 (CCNA2), CDC20, CDC45, MMP1, MMP3, MMP9, MMP10, STAT1, STAT2, TNFRSF1A, TNFSF10, and TUBB3 were significantly upregulated in bladder cancer cases. We showed that biological reactions, including the degradation of collagen and extracellular matrix and matrix metalloproteinases activation reactions, were found the most statistically significant pathways in BC. We also determined that inflammation and cytokine signalling could be related to the progression of bladder carcinogenesis. The genes differentially expressed could be molecular indicators for early prediction of bladder carcinogenesis.

**Supplementary Information:**

The online version contains supplementary material available at 10.1038/s41598-026-36530-1.

## Introduction

 Globally, bladder cancer (BC) ranks as the ninth most commonly diagnosed cancer^1^. Nearly 75% of newly diagnosed BC patients have non-invasive cancer types, while others have muscle-invasive or advanced metastatic cancer types^2^. Though the non-muscle-invasive type is the most commonly seen BC type, it has the potential to progress muscle-invasive type^3^. Early diagnosis and multimodality therapy result in optimal patient outcomes; the metastatic disease is generally incurable, with a relative 5-year^4^. In addition to surgery, chemotherapy, and radiotherapy, immunotherapy has emerged as a standard treatment option and has been incorporated into the National Comprehensive Cancer Network (NCCN) guidelines since 2021, particularly for patients with advanced or metastatic BC^5^. In parallel with these advances, mutation-targeted therapies have emerged in selected molecular subgroups of bladder cancer. For example, erdafitinib, a pan-FGFR inhibitor, has been approved for the treatment of advanced or metastatic bladder cancer with FGFR2/3 alterations, underscoring the clinical relevance of molecularly driven therapeutic strategies^6^. Despite these advances, the molecular mechanisms underlying BC pathogenesis are not yet fully elucidated^7^.

In molecular oncology, tumor characterization has traditionally relied on the identification of genetic variants, which are commonly classified as pathogenic, likely pathogenic, benign, or variants of uncertain significance (VUR)^8^. While pathogenic variants are directly implicated in carcinogenesis, VUS remain challenging to interpret due to their unclear functional consequences^9^. Importantly, dysregulated gene expression may reflect downstream functional effects of both pathogenic variants and regulatory or epigenetic alterations that are not captured by variant-level analyses alone. Therefore, transcriptome-wide expression profiling provides complementary insight by highlighting quantitatively altered genes and pathways associated with tumor aggressiveness and progression.

BC is strongly influenced by environmental and lifestyle-related risk factors. Tobacco smoking, occupational exposure to carcinogenic chemicals, and chronic bladder inflammation are known to induce DNA damage and genomic instability, leading to genetic and transcriptional alterations^10^. These factors may act synergistically to promote tumor initiation and progression, highlighting the importance of investigating downstream gene expression changes associated with aggressive BC.

Most of the epithelial cancer cells transform from benign cell to malignant cell through mutations in essential genes that modulate vital cellular bio functions such as proliferation, migration and apoptosis^11^. Epithelial–mesenchymal transition (EMT) stimulates angiogenesis and metastasis in the tumorigenic process by showing various clinicopathological characteristics^12,13^. Dysregulation of the cell cycle emerges as the most defined cellular process involving bladder carcinogenesis^14–16^. Cell migration between biological compartments is the most critical step for the invasion process during tumorigenesis^17–19^. Besides, proteolytic enzymes’ secretion, causing the degradation of ECM proteins, may disrupt cell–cell interactions during metastatic progression^17–19^. MMPs are the most remarkable zinc-dependent endopeptidases involved in the metastatic process during carcinogenesis^20^. The MMP family members could include in the metastatic process, such as MMP1, MMP2, and MMP7 and MMP9, which are the most significant genes throughout cellular migration and the urothelial cell invasion carcinoma of the bladder (UCB). In this study, we aimed to identify specific gene-pathway relationships that could play a fundamental role in the progression of bladder carcinogenesis.

## Materials methods

Twenty patients who underwent transurethral resection of bladder tumor (TUR-BT) with a preliminary diagnosis of primary bladder cancer were included in the study. Each patient was interviewed before surgery and detailed information was given about the research. Written informed consent was obtained from all the participants during their enrolment for the study. During the operation, a sample of the macroscopically visible tumor tissue and a sample of the area where normal mucosa was visible were removed. Samples (*n* = 20) of bladder cancer patients and their non-tumoral paired controls (*n* = 20) were collected. Paired controls from non-tumoral surrounding tissue were taken from the clinically unaffected region and histologically controlled. Tumor samples were pathologically staged according to the rules of AJCC (American Joint Committee on Cancer). Tissue samples were saved on RNA later solution to prevent RNA degradation and moved to the Department of Biochemistry, Istanbul University. Criteria for inclusion in the study: being over 18 years of age, no previous bladder-related surgery, no other cancer. Low-grade tumors and cases with variant histology were excluded to reduce biological heterogeneity and to focus on high-grade conventional urothelial carcinoma. A total of eight participants’ samples were excluded from the study because the pathological examination of three samples was low grade, three samples had variant histology, a sample could not be isolated with sufficient RNA, and a sample was detected as carcinoma in situ in normal tissue. Samples from a total of 12 participants were included in the study. Ethical approval was approved by the Istanbul Faculty of Medicine Ethics Committee. (Number: 1037-337). All experimental procedures and protocols were conducted in full accordance with the ethical standards and relevant guidelines and regulations established by institutional and national research committees, and in line with the principles of the Declaration of Helsinki. This study was supported by Istanbul University Scientific Research Projects (Project number:16206).

### RNA extraction

RNA isolation was performed from tumoral and non-tumoral tissue samples using High Pure Tissue Isolation Kit due to the manufacturer’s instructions following the homogenization process. After measuring the quality and quantity of RNA samples using Nanodrop (Thermo-Scientific, USA), the integrity of RNA samples was also determined using the Agilent 2100 bio-analyzer (Santa Clara, CA).

### Target sample generation

The biotinylated and amplified RNA, which was used in turn hybridization step, was constituted with The Illumina TotalPrep RNA Amplification Kit according to instructions. Briefly, an oligo (dT) primer that consisted of a T7 promoter was generated with Array Script to improve the efficiency of cDNA yield. The cDNA samples were used in vitro transcription by T7 RNA Polymerase as a template to synthesize cRNA. In the last step, cRNA (500 ng) samples were fragmented and then inserted into the hybridization solution.

### Array hybridization

The expression profiles of the whole human genome were determined using Illumina Human HT-12V4 microchips that targeted more than 47,000 probes from NCBI Release 38 and other sources. The hybridization processes were applied according to the instructions of Illumina. The bead chips were scanned on the Illumina Iscan system. Background signal intensities of probe sets were corrected using quantile normalization. The boxplots were shown before and after normalization (Supplementary Fig. 1). Density plots were indicated after normalization (Supplementary Fig. 2).

### Data analysis

The signaling intensities of each sample that corresponded to gene expression levels were corrected and normalized using Illumina Genome Studio Gene Expression Module. In the next step, data sets were hierarchically clustered to measure the proximal distance between statistically significant genes. The hierarchic clustering method was performed on the differentially expressed genes set to find whether distinct conditional groups could be categorized with statistically significant genes. The heatmap analysis demonstrated that all primary tumors and control tissue samples were clustered together in their different conditional groups. Furthermore, an adjusted *p* value of these genes was computed to define significantly different genes that had more than a 1.5-fold change ratio and *p* value under 10^−3^. Pathway and network analyses were performed using Ingenuity Pathway Analysis (IPA; QIAGEN, URL: https://www.qiagen.com/us/products/discovery-and-translational-research/next-generation-sequencing/informatics-and-data/interpretation-content-databases/ingenuity-pathway-analysis) and Cytoscape software (version 3.9.0, URL: https://cytoscape.org) to identify statistically significant genes, interaction networks, and biological pathways differentiating tumor and control samples.

## Results

We determined the distinction of gene expression patterns between high-grade bladder cancer patients (*n* = 12) and their paired control (*n* = 12) tissue samples from clinically unaffected regions. Three of the participants were women and nine were men. The pathology results of three of them were reported as pT2 and nine of them were reported as pT1.

Due to the core analysis results, the most statistically significant bio-functions were post-translational modification, protein degradation, cell death and cell survival, cellular movement, and intercellular signaling (Supplementary Table 1). These findings supplied us an understanding of which molecular reactions were involved in bladder carcinogenesis. Regarding these results, we also identified the top canonical pathways included in the development of BC (Table 1). The data showed that collagen degradation, activation of MMPs, degradation of the ECM, inflammatory response and BC signaling were the most critical pathways in bladder carcinogenesis, as expected. Furthermore, MMP1, MMP3, MMP9 and MMP10 genes were found to be the most relevant genes that were connected with the progression of bladder cancer (Table 1).

**Table 1:** Top canonical pathway analysis.

Pathway name	#Entities	*p* value	Submitted entities hit interactor
Collagen degradation	6	7.40E^−10^	MMP9
Activation of matrix metalloproteinases	5	1.80E^−08^	MMP9
Bladder cancer signaling	4	8,47E^−08^	MMP1; MMP3; MMP9; MMP10
Degradation of the extracellular matrix	6	2.03E^−07^	MMP9
Cytokine signaling in immune system	8	7.09E^−06^	CCNA2; STAT1; MMP1; STAT2; MMP9
Signaling by interleukins	6	8.84E^−06^	CCNA2; MMP1; STAT1; MMP9
Cellular responses to stress	2	8.49E^−05^	CCNA2; CDC45; MMP1; STAT1; MMP9
Cellular responses to external stimuli	2	1.69E^−04^	CCNA2; CDC45; MMP1; STAT1; MMP9
Immune system	11	1.95E^−04^	CCNA2; STAT1; MMP1; STAT2; MMP9
Interferon alpha/beta signaling	3	5.77E^−04^	STAT1; STAT2
Cell cycle, mitotic	4	9.63E^−04^	CCNA2; CDC20; CDC45; STAT1
Cell cycle checkpoints	3	0.002	CDC20; CCNA2; CDC45
Mitotic G1-G1/S phases	2	0.005	CCNA2; CDC45; STAT1
Cell cycle	4	0.007	CCNA2; CDC20; CDC45; STAT1

**Fig. 1:**
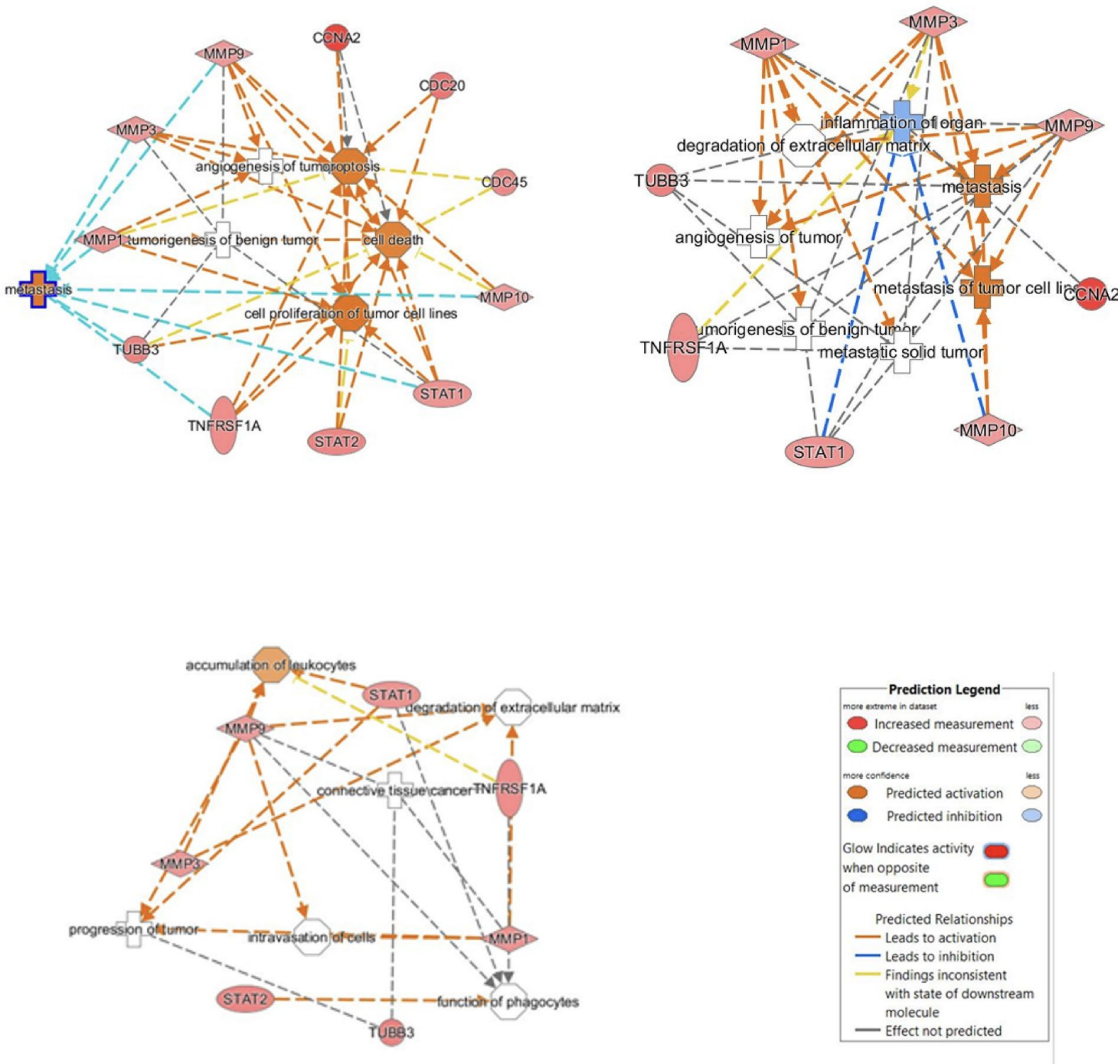
Gene annotation analysis: according to gene ontology analysis, we showed that cell proliferation, angiogenesis, tumorigenesis, and degradation of matrix proteins were related with genes upregulated in microarray data.

According to diseases and disorders analysis, cancer, inflammatory response, and immunodeficiency were associated with differentially expressed gene patterns, respectively (Supplementary Table 2). We also clustered these genes with gene ontology analysis and indicated that invasion, metastasis, neoplasm, tumorigenesis, and angiogenesis were associated with inflammation and degradation of the matrix in support of the progression of bladder cancer (Fig. 1). In addition, two main pathway groups appeared to be necessary for the BC, including cell cycle, cancer, reproductive system disease (score = 32), and also cell signaling/ interaction, inflammatory response (score = 30) through network analysis (Supplementary Table 3).

**Fig. 2:**
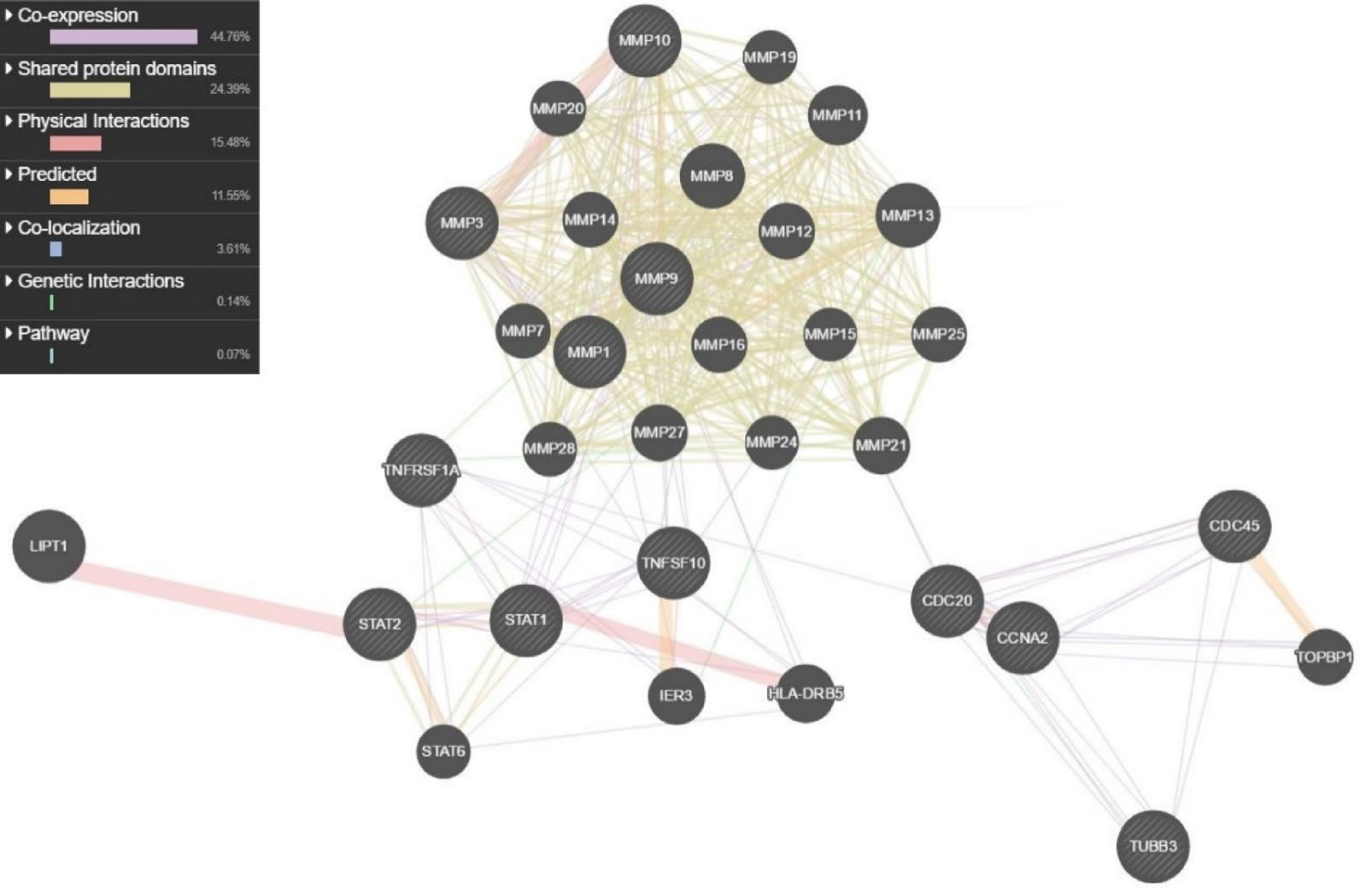
Gene network analysis: we showed the interaction of genes which were significantly upregulated in our microarray data as a network map.

The interconnection between significant genes that were involved in top network analysis was demonstrated in Fig. 2. Due to the microarray data, it was found that the expression levels of MMP1, MMP3, MMP9, MMP10, STAT1, STAT2, CCNA2, TUBB3, CDC20, CDC45, TNFRSF1A, TNFSF10 (TRAIL) genes were significantly increased in bladder cancer patients compared with paired controls as shown in Table 2. It was also detected that these top-upregulated molecules were the most statistically significant genes involved in all essential biological processes, such as metastasis, invasion, and angiogenesis which were related to bladder carcinogenesis (Fig. 1, Supplementary Table 4).

**Table 2:** Fold-change upregulated top Molecules.

Gene symbol	Fold change	*p* value
Cyclin A	3.018	1.93E^−04^
CDC20	2.105	2.48E^−04^
CDC45	1.807	1.35E^−03^
MMP1	1.649	1.22E^−04^
MMP3	1.586	1.16E^−04^
MMP9	1.545	1.96E^−04^
MMP10	1.537	1.78E^−04^
STAT1	1.638	1.75E^−03^
STAT2	1.849	1.95E^−03^
TNFRSF1A	1.742	2.23E^−03^
TNFSF10	1.864	2.03E^−03^
TUBB3	1.917	2.96E^−07^

## Discussion

In this study, our goal was to determine gene-gene interactions and pathways involved in bladder carcinogenesis to find new biomarkers for targeted cancer therapy. Based on microarray data, we found that the expression levels of MMP1, MMP3, MMP9, MMP10, STAT1, STAT2, CCNA2, TUBB3, CDC20, CDC45, TNFRSF1A, TNFSF10 (TRAIL) genes were significantly upregulated in bladder cancer patients compared with their paired controls. Results of gene ontology analysis a; so, showed us that these genes were involved in the most crucial steps in the progression of bladder carcinogenesis, such as angiogenesis, hyperplasia, invasion, and metastasis.

Matrix metalloproteinases, which are known as a family of zinc-dependent endopeptidases, contribute to various pathological conditions such as cancer development, atherosclerosis, and osteoarthritis^17–20^. MMPs play an essential role in the degradation of extracellular matrix proteins to promote tumor invasion and metastasis^21^. Upregulation in the MMP gene family was reported to include invasive and metastatic processes of bladder carcinogenesis^22^. Chuang et al. demonstrated that MMP-1 expression was correlated with advanced BC^17^. Wieczorek et al. found that MMP-9 gene expression was statistically increased in peripheral blood leukocytes of moderately differentiated BC patients^18^. Additionally, Candido et al. showed that MMP-9 played an essential role in the main events included in the metastatic process of bladder carcinogenesis^20^. Our results demonstrated that the upregulation of MMP1, MMP3, MMP9 and MMP10 genes was related to bladder cancer in line with previous studies.

STATs (signal transducers and activators of transcription) consist of latent transcription factors that generally participate in cell growth, survival, and differentiation. The activation of STAT proteins occurs through a series of events, including phosphorylation of tyrosine residue with Janus-activated family kinases (JAK) following cytokine, growth factor and hormone induction^23,24^. In a study, the upregulation of STAT genes has been reported to involve in the tumor cell transformation process^25^. Sun et al. suggested that muscle-invasive BC was characterized by elevated expression levels of pSTAT1, pSTAT3 and pSTAT5^23^. In addition, it was also indicated that inhibition of the STAT pathway could decrease the invasiveness of bladder carcinogenesis. Furthermore, Itoh et al. showed that STAT3 phosphorylation was required for the induction of MMP genes in response to epidermal growth factor on T24 bladder cancer cell lines^24^. Our findings were correlated with previous reports that showed upregulated expressions of the STAT gene family in bladder carcinogenesis.

Microtubules are cytoskeletal proteins formed by the polymerization of α and β-tubulin subunits that participate in cell shaping, intracellular transport and chromosome segregation events^26^. TUBB3 (class III β-tubulin) is a β-tubulin isotype that has been indicated to stimulate tumor invasiveness in various cancer types such as ovarian, breast, lung, prostate, renal and bladder cancers^26,27^. Our results were in agreement with literature showing overexpression of TUBB3 in urinary BC^27^.

The cell division cycle is firmly regulated by the anaphase promoting complex/cyclosome complex (APC/C), which is a well-known ubiquitin ligase activating by Cdc20 or Cdh1^28,29^. Hein et al. indicated that phosphorylation of Cdc20 with CCNA2-Cdk2 complex was required to suppress APC/C activation for effective mitotic entry^28^. As a result of impaired activation of the APC/C complex, accumulation of CCNA2 may lead to proliferation, migration and invasion of the carcinogenesis. Previous studies showed that overexpression of Cyclin A2 was related to the progression of various human carcinomas^30^. Aberrant expression of CDC20 was also suggested to impair activation of the APC/C complex, which led to mitotic abnormalities in many types of tumors, including BC. Our results indicated that Cyclin A2 and Cdc20 upregulation was associated with high grade bladder tumors. Therefore, CCNA2 and CDC20 may be used as biomarkers to predict high-grade BC patients.

CDC45 is a protein part of the eukaryotic replicative helicase CMG complex, including CDC45, Mcm2-7 and GINS. Cdc45 was proposed to activate dormant origins in initiation and elongation steps during the cell division cycle S phase^31^. Previous studies showed that elevated expression of Cdc45 was tightly related to the proliferation of cancer cells to create many origins in the replication fork for early S phase arrest^31,32^. We also found that overexpression of Cdc45 was associated with the progression of BC in agreement with other cancer studies.

TNF ligands are shown to modulate inflammation and cell survival by binding to two different cell surface receptors (TNFRI or TNFR2)^32^. TNFRI activates apoptotic signal-regulating kinase I that mediates cell death. However, TNFR2 stimulates endothelial tyrosine kinase signaling that transactivates vascular endothelial growth factor receptor 2 (VEGFR2) to promote cell proliferation^32^. Although both TNFRI and TNFR2 are demonstrated to play essential roles in tumor progression, the mechanism of TNFRI action is better understood. The overexpression of TNFRI could contribute to tumorigenesis via stimulation of the p42/44 MAPK, JNK and PI3K/Akt pathways^33^. Here, we also reported that the increased TNFRI expression was correlated with bladder carcinogenesis progression. Thus, TNFRI overexpression has been suggested to be an essential indicator of the development and progression of bladder cancer patients. Autophagy is an essential intracellular process involving the degradation of long-lived proteins and organelles to regulate homeostasis. Under cellular stress, such as nutrient starvation, autophagy is quickly upregulated to supply enough energy for cell survival. However, autophagy can contribute to PCD II (type II programmed cell death), which is morphologically different from apoptosis in certain circumstances^34^. TNFSF10/TRAIL, which belongs to the TNF superfamily, has been shown to stimulate autophagy-mediated cell survival in specific cancer cells, but its mechanism of action is poorly understood^35^. Furthermore, the effect of autophagy on the regulation of cell death control is also contradictory because of its protecting or promoting role in therapy-stimulated cell death^36^. In this study, we found that TNFSF10/TRAIL was overexpressed in high-grade bladder cancer patients.

In other malignancies such as breast and prostate cancer, genetic profiling has already been successfully integrated into population-based screening and risk stratification programs, enabling personalized surveillance and early detection strategies. These examples highlight the increasing role of genomics in precision oncology and provide a relevant framework for interpreting transcriptomic findings in bladder cancer.

Several limitations of this study should be acknowledged. First, the relatively small sample size reflects the challenges of obtaining high-quality paired tumor and non-tumoral bladder tissues for genome-wide transcriptomic analysis and may limit the generalizability of our findings. Second, the cross-sectional design does not allow assessment of temporal changes in gene expression or direct correlations with long-term clinical outcomes such as recurrence, progression, or survival. Third, although our integrative pathway and network analyses provide biological insight, the findings were not validated in an independent external cohort, nor were functional experiments performed to confirm the mechanistic roles of the identified genes. Finally, the study focused exclusively on high-grade conventional urothelial carcinoma, and therefore the results may not be directly applicable to low-grade tumors or variant histologies.

Our finding is the first research that simultaneously demonstrated the expression changes for CDC20, CDC45, CCNA2, STAT1, STAT2, TNFRI and MMPs in high-grade bladder cancer patients. Although we found new biomarkers for the development of BC, the case-control number is the limitation of our study. Future researches are necessary to understand molecular pathways related to the mechanism of action underlying the progression of bladder carcinogenesis. We strongly believe that this study’s results will guide future research and help develop new biomarkers for early prediction and increasing survival ratios.

## Supplementary Information

Below is the link to the electronic supplementary material.

Supplementary Material 1

## Data Availability

The datasets generated and/or analysed during the current study are available from the corresponding author upon reasonable request.
