# Supplementary material for: Genome-wide transcriptional profiling identifies molecular markers associated with early carcinogenesis in high-grade bladder cancer
Source: Sci Rep. 2026 Mar 26;16:15150. doi: 10.1038/s41598-026-36530-1 (PMC13172329; doi:10.1038/s41598-026-36530-1)
Supplement: Supplementary file 1 — Supplementary Material 1 [file 41598_2026_36530_MOESM1_ESM.docx]

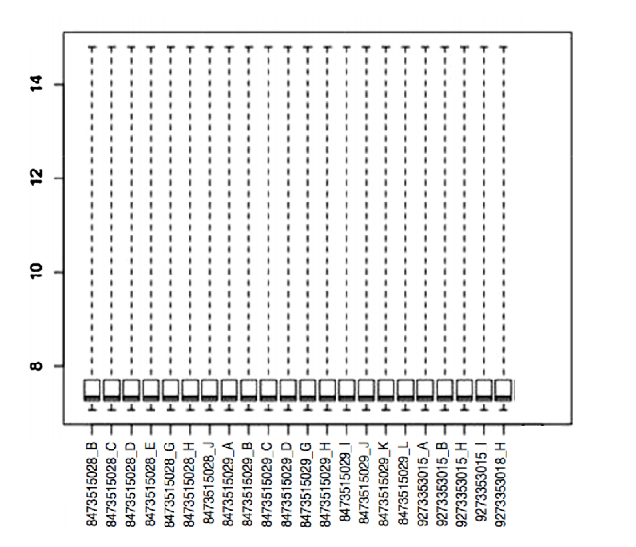

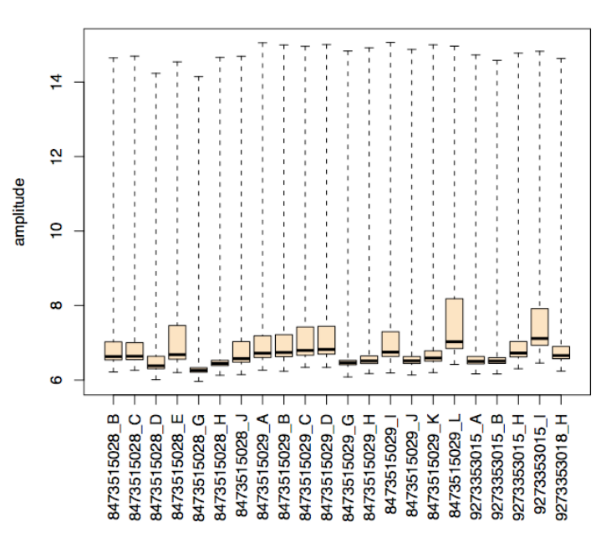


**Supplementary Figure 1:** Box-plots before (a) and after (b) normalization (tumor and control samples were grouped to perform differential expression analysis with obtained data from normalization).


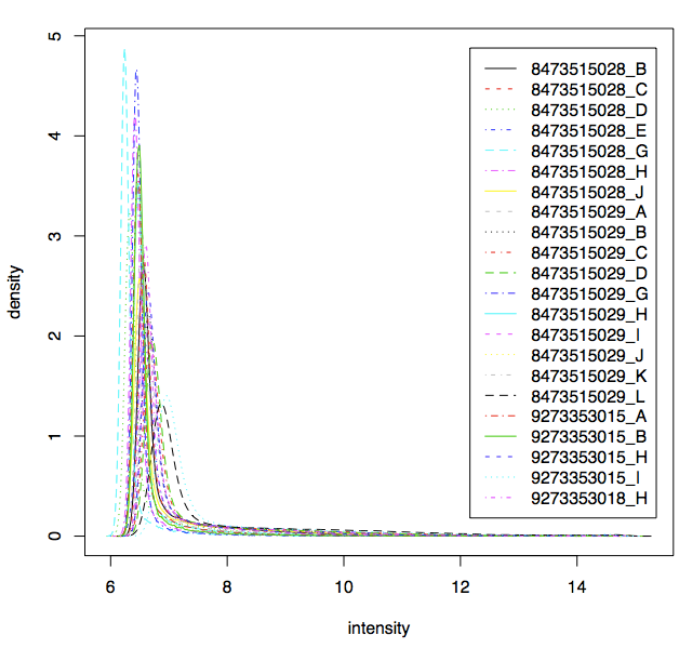


**Supplementary Figure 2:** Density plot (tumor and control samples were grouped to perform differential expression analysis with obtained data from normalization)

| **Molecular and Cellular Functions** | **p-value** | **#Molecules** |
| --- | --- | --- |
| Post-Translational Modification | 2,87E^-4^ – 5,78E^-10^ | 4 |
| Protein Degradation | 2,87E^-04^ – 5,78E^-10^ | 4 |
| Cell Death and Survival | 2,61E^-03^ – 1,25E^-07^ | 11 |
| Cellular movement | 2,16E^-03^ – 8,76E^-06^ | 8 |
| Cell to cell signaling signaling and interaction | 3.25E^-04^ to 1.29E^-04^ | 5 |

**Supplementary Table 1:** Molecular and Cellular Biofunctional Analysis

| **Diseases and disorders** | **p-value** | **#Molecules** |
| --- | --- | --- |
| Cancer | 1.59E^-03^ to 5.62E^-03^ | 10 |
| Immunological disease | 1.48E^-03^ to 4.35E^-03^ | 6 |
| Inflammatory response | 1.23E^-03^ to 3.20E^-0.3^ | 3 |

**Supplementary Table 2:** Top Diseases and Disorders Analysis

| **Molecular Functions and Top Diseases** | **p-value** | **Score** | **Molecules in Network** |
| --- | --- | --- | --- |
| Cell cycle, cancer, reproductive  system disease | 1.08E^-04^ to 1.14E^-04^ | 32 | CCNA2, CDC20, CDC45,  STAT1, STAT2, TUBB3 |
| Cell-to-cell signaling and interaction,  inflammatory response | 3.25E^-04^ to 1.29E^-04^ | 30 | MMP1, MMP3,MMP9,MMP10,  TNFSF10, TNFRSF1A |

**Supplementary Table 3:** Network Analysis

| **Diseases or Functions Annotation** | **p-Value** | **Molecules** | **# Molecules** |
| --- | --- | --- | --- |
| metastasis | 5,03E^-07^ | MMP1, MMP10, MMP3, MMP9, STAT1, TNFRSF1A, TUBB3 | 7 |
| neoplasia of tumor cell lines | 1.43E^-06^ | MMP1, MMP10, MMP3, MMP9, STAT1 | 5 |
| metastasis of tumor cell lines | 5,26E^-06^ | MMP1, MMP10, MMP3, MMP9 | 4 |
| progression of tumor | 1.65E^-05^ | MMP1, MMP9, STAT1, TUBB3 | 4 |
| incidence of tumor | 3.86E^-05^ | MMP1, MMP9, STAT1, TNFRSF1A | 4 |
| angiogenesis of tumor | 4.35E^-05^ | MMP1, MMP3, MMP9 | 3 |
| invasion of tumor cells | 1,40E^-04^ | MMP1, MMP3, MMP9 | 3 |
| progression of malignant tumor | 1,50E^-04^ | MMP1, MMP9, T UBB3 | 3 |
| growth of malignant tumor | 1,57E^-04^ | MMP1, MMP3, MMP9, STAT1 | 4 |
| growth of tumor | 1,68E^-04^ | MMP1, MMP3, MMP9, STAT1, TUBB3 | 5 |
| invasion of tissue | 1,89E^-04^ | MMP1, MMP3, MMP9 | 3 |
| hyperplasia of tissue | 2,20E^-04^ | CCNA2, MMP1, MMP9 | 3 |
| cancer of cells | 5,43E^-04^ | CDC45, MMP1, MMP10, MMP3, MMP9, STAT1, TNFRSF1A, TUBB3 | 8 |
| invasion of epithelial tissue | 6,28E^-04^ | MMP1, MMP9 | 2 |

**Supplementary Table 4:** Gene ontology analysis
